# A Short-Term Response of Soil Microbial Communities to Cadmium and Organic Substrate Amendment in Long-Term Contaminated Soil by Toxic Elements

**DOI:** 10.3389/fmicb.2018.02807

**Published:** 2018-11-20

**Authors:** Pavla Madrova, Tomas Vetrovsky, Marek Omelka, Michal Grunt, Yvona Smutna, Daria Rapoport, Marek Vach, Petr Baldrian, Jan Kopecky, Marketa Sagova-Mareckova

**Affiliations:** ^1^Department of Epidemiology and Ecology of Microorganisms, Crop Research Institute, Prague, Czechia; ^2^Laboratory of Environmental Microbiology, Institute of Microbiology of the AS CR, v.v.i., Prague, Czechia; ^3^Department of Probability and Mathematical Statistics, Faculty of Mathematics and Physics, Charles University, Prague, Czechia; ^4^Department of Water Resources and Environmental Modeling, Faculty of Environmental Sciences, Czech University of Life Sciences, Prague, Czechia; ^5^Department of Microbiology, Nutrition and Dietetics, Faculty of Agrobiology, Food and Natural Resources, Czech University of Life Sciences, Prague, Czechia

**Keywords:** respiration, diversity, actinobacteria, straw, cellobiose

## Abstract

Two long-term contaminated soils differing in contents of Pb, Zn, As, Cd were compared in a microcosm experiment for changes in microbial community structure and respiration after various treatments. We observed that the extent of long-term contamination (over 200 years) by toxic elements did not change the total numbers and diversity of bacteria but influenced their community composition. Namely, numbers of *Actinobacteria* determined by phylum specific qPCR increased and also the proportion of *Actinobacteria* and *Chloroflexi* increased in Illumina sequence libraries in the more contaminated soil. In the experiment, secondary disturbance by supplemented cadmium (doses from double to 100-fold the concentration in the original soil) and organic substrates (cellobiose or straw) increased bacterial diversity in the less contaminated soil and decreased it in the more contaminated soil. Respiration in the experiment was higher in the more contaminated soil in all treatments and correlated with bacterial numbers. Considering the most significant changes in bacterial community, it seemed that particularly *Actinobacteria* withstand contamination by toxic elements. The results proved higher resistance to secondary disturbance in terms of both, respiration and bacterial community structure in the less contaminated soil.

## Highlights

– Bacterial communities differed between highly (H) and low (L) contaminated soils in proportion and quantity of *Actinobacteria* but the number of total bacteria was similar.
– *Actinobacteria* increased after addition of cadmium and organic substrates in both soils.
– Respiration increased more in the H soil after addition of cellobiose and straw.
– The H soil was more sensitive to additional disturbance than the L soil.

## Introduction

Ecosystem health has been defined in terms of stability and resilience in response to disturbance (Griffiths and Philippot, 2013). The amplitude of this response and time to return to the state before disturbance can serve as measures of soil health (Van Bruggen and Semenov, 2000). Many relationships in soil microbial communities are accompanied by functional redundancy, which allows conservation of important ecosystem functions in spite of changes in microbial community (Singh et al., 2014). Therefore, it seems that composition of soil bacterial community represents a sensitive measure of soil health compared to general soil characteristics such as respiration or other measures of organic matter decomposition (Hartmann et al., 2014; Singh et al., 2014).

Pollution by toxic elements represents a chemical disturbance of worldwide concern. It causes major changes to soil functioning including decomposition of organic matter or element cycling (Azarbad et al., 2013; Li et al., 2014). However, this type of disturbance can be counterweighted by adaptation of microbial communities to the contamination. Mostly because microorganisms were exposed to toxic elements since early history, which resulted in the evolution of metal resistance mechanisms, such as extracellular and intracellular sequestration of metals, exclusion by permeability barriers, enzymatic detoxification, reduction in sensitivity of cellular targets or efflux pumps (Epelde et al., 2015). Although metal toxicity to soil microorganisms leading to changes of their activities had been confirmed, it remained unclear whether they resulted from an overall decrease in microbial diversity or rather from inhibition of specific taxa. Also it was shown that different activities are unevenly affected (Niemeyer et al., 2012). With respect to that it was hypothesized that organic matter breakdown, as one of the general microbial functions, may be relatively unchanged because most microorganisms utilize different forms of organic carbon. However, respiration rates as a measure of decomposition were found both positively or negatively related to toxic elements contamination (Niemeyer et al., 2012; Epelde et al., 2015).

Actinobacteria represent a specifically interesting group for adaptation strategies in decomposition processes (Sagova-Mareckova et al., 2011). They are not only one of dominant groups of soil bacteria but they belong to the most efficient decomposers breaking down even hemicellulose and lignin. Above that it was found that actinobacteria are more sensitive to toxic elements contamination than fungi and bacteria in general, so it seems that they have a potential to become bioindicators of this type of disturbance (Jin et al., 2014).

One specific situation of toxic elements pollution is represented by metal smelters, around which the soil was unpolluted before smelting started and then, over decades, have become highly contaminated. Therefore, soil microbial communities in such areas became adapted to metal contamination. New metal-tolerant microbial communities developed due to selection pressure and horizontal gene transfer of respective resistance genes so the soil functions were maintained (Azarbad et al., 2016). However, due to constant use of the contaminated land and possibly also the climate change, new changes of environmental conditions occur at contaminated areas. Those may be characterized as secondary disturbance and may include changing the land use for other type of crops, forest or meadow, fertilization, fire or flooding or invasion by a foreign organism. Not much is known about the outcome of such secondary disturbance in terms of microbial community structure or functions, in particular, when the effect of several disturbing factors is combined (Azarbad et al., 2016).

We tested the effect of newly added cadmium and organic substrates to see, which parameter out of respiration, quantity of total bacteria and actinobacteria and bacterial community structure will correspond to induced disturbance and how that will compare between two soils differing in the extent of long term contamination with toxic elements. Cadmium as a contaminating metal was selected because of its high toxicity and also because previous results obtained at the same site showed that Cd concentration was significantly correlated to respiration rate and metabolic quotient, parameters closely related to activities of microorganisms (Muhlbachova et al., 2015). The first organic substrate, cellobiose was selected because it is a common disaccharide, which is produced from cellulose by cellobiohydrolase. It is an easily utilized substrate, preferably consumed and may be connected to decomposition processes occurring in the root zone, which is considered a decomposition hotspot (Steinauer et al., 2016). Variability of cellobiohydrolase gene was compared in the two soils. This enzyme was selected as an example of a specific microbial function related to decomposition process. The second organic substrate, straw was chosen for its high content of lignin, which is the least degradable part of organic matter, therefore its decomposition is slower. The detailed analysis of changes in microbial community structure in the treatments using real-time PCR and Illumina amplicon sequencing was performed to show how changes in respiration are related to bacterial community. A specific attention was given to actinobacteria because of their known large pool of resistance genes. We hypothesized that the more contaminated soil will have a stronger response to additional disturbance than the less contaminated soil and that different taxonomic groups will, respectively, change their relative abundance to applied amendments in each soil. Those taxa can then serve as microbial indicators for responding to long-term and short term toxic elements contamination.

## Materials and Methods

### Sites

The sampling sites were located in an area contaminated with toxic elements near a lead smelter in Příbram, Czechia. Two grasslands (grass planted in old fields), H (site with high in concentration, N49°42.327 E13°58.516) and L (site with low concentration, N49°42.243 E13°56.371) were selected for this study (Table 1). The soil at both sites is slightly acidic modal Cambisol. The more contaminated site was about 300 m, while the less contaminated site about 3,000 m apart from the smelter chimney, which represents the major source of deposits. They differed in contamination not only due to differing distance but also due to prevailing northwest winds. The smelter processed lead ores from 1786 to 1972, when processing of secondary lead sources started. Since 1982 emissions of contaminating elements decreased 300–500 times. Contamination of the area and explanations were previously described in Muhlbachova et al. (2015). Soil was sampled November 2013. At each site, five soil cores (40 mm diameter) were taken from the upper soil horizon to depth of approximately 150 mm. Cores were collected randomly from the area of 4 m^2^ using plastic tubes. Soil of each core after removal of grass rhizosphere was homogenized through a 2-mm sieve to produce subsamples for subsequent experiment and analyses.

**Table 1:** Characteristics of sites with high (H) and low (L) soil contamination.

	H	*SD*	L	*SD*
pH	5.40	0.10	5.40	0.10
Organic matter	4.72%	0.23	5.3%	0.32
Pb ^∗∗∗^	451.3 mg.kg^-1^	48.30	65.6 mg.kg^-1^	6.25
Zn ^∗∗∗^	180.6 mg.kg^-1^	7.00	78.9 mg.kg^-1^	4.93
As ^∗∗∗^	30.41 mg.kg^-1^	9.86	13.517 mg.kg^-1^	1.02
Cd ^∗∗∗^	15.055 mg.kg^-1^	0.66	8.307 mg.kg^-1^	0.37

*^∗∗∗^*p* < 0.001, *n* = 5.*

### Short-Term Experiment

Firstly, soils collected at the two sites were compared in terms of microbial communities using a subsample (0.5 g) from each core for DNA extraction and downstream applications. Those samples are named “original soils” and represent the long term change of the two differently contaminated sites. Secondly in a short term experiment, subsamples of 15 g of the respective cores were used in OxiTop respiration measurements. Those subsamples were treated with cadmium, cellobiose and straw according to Table 2. The treatments also included a “treatment” without supplements, which was subjected to respiration measurements. Dosages of cadmium were derived from its contents in the original soils (Table 1). The lowest dosage doubled the average original content, the following dosages were 10- and 100-fold the original content. Soil was collected after exposure in OxiTop flasks and stored at -80°C until further processing. we chose to determine the first phase of the microbial community response to toxic elements and organic supplements because we had observed previously that in microcosms respiration rate is usually consistent only during the first 5–7 days (Li et al., 2014). Finely grated wheat straw and D-cellobiose (Sigma-Aldrich) 3 g each were added to respective treatments.

**Table 2:** Sample treatments in the short-term experiment applied for both the low contamination soil L and the high contamination soil H.

Sample number	Cadmium (mg.kg^-1^)	Substrate
0	0	-
10	10	-
100	100	-
1000	1000	-
C0	0	Cellobiose (C), 3 g
C10	10	Cellobiose (C), 3 g
C100	100	Cellobiose (C), 3 g
C1000	1000	Cellobiose (C), 3 g
S0	0	Straw (S), 3 g
S10	10	Straw (S), 3 g
S100	100	Straw (S), 3 g
S1000	1000	Straw (S), 3 g

### Respiration

Soil microbial respiration was measured by a pressure sensor method using OxiTop system with pressure sensor-data logger OxiTop-C heads including a capsule for NaOH solution (WTW, Weiheim, Germany). The respiration rate was calculated from linear pressure decrease equivalent to O_2_ consumption between 50 and 250 h of incubation.

### Soil Analysis

Soil samples were analyzed for pH, organic matter content, lead, zinc, arsenic, and cadmium contents. Soil pH was measured by a Multi 350 glass electrode WTW, in a soil-water extract, 20 g of soil being extracted with 50 ml distilled water, and set at room temperature for 12 h. Organic matter was assessed by combustion at 550°C to constant weight. the leachable toxic elements (Pb, Zn, As, Cd), were determined in 5 g dry soil samples extracted with 50 ml 2 M nitric acid after 6 h shaking (Suprapur; Merck, Darmstadt, Germany). The filtered extract was analyzed using atomic absorption spectrometry (AAS). Determination of Cd and Pb was performed by atomizing in a graphite tube with a 240Z AA device with Zeeman background correction (Agilent Technologies, Santa Clara, CA, United States). Zn and As were determined by flame AAS on the 55 AA device, and arsenic determination was performed using Vapor Generation Accessory VGA 77 (Agilent Technologies).

### Soil DNA Extraction

DNA was extracted from 0.5 g of soil samples by the method described in Sagova-Mareckova et al. (2008), where it is denoted “SK.” The method is based on bead-beating and phenol/chloroform extraction. The samples are purified by incubation with cetyltrimethylammonium bromide followed by chloroform extraction and incubation with CaCl_2_, and finally cleaned with GeneClean Turbo kit (MP Biomedicals, Santa Ana, CA, United States). DNA quality and quantity was estimated using an agarose gel electrophoresis and the concentration was measured by NanoPhotometer (Implan, Germany).

### PCR Amplification and Cloning of Bacterial Cellobiohydrolase Gene

Subsamples of two randomly selected biological replicates from controls and treatments with 100 mg/kg Cd and straw were selected for cellobiohydrolase gene sequencing. Primers Cbh1F (5′- CGTCRTCTACRACCTGCC -3′) and Cbh2R (5′- CCAGCCGAKCCAGCCGTG -3′) were designed using Primrose software included in Bioinformatic toolkit (Ashelford et al., 2002) based on the known cellobiohydrolase encoding genes retrieved from the GenBank database. The primers targeting a 346 bp part of bacterial cellobiohydrolase gene were validated by amplification and cloning from *Nocardiopsis dassonvillei* subsp. *dassonvillei* chromosomal DNA and from environmental DNA samples isolated at the study sites. The primers were designed to amplify cellobiohydrolase genes from *Actinobacteria*. According to the Carbohydrate-active enzymes database^1^ (Lombard et al., 2014) the primers cover cellobiohydrolases cleaving from non-reducing end (EC3.2.1.91) classified in glycosyl hydrolases class 6 and do not amplify the genes coding for endoglucanases (EC 3.2.1.4) of the same class. PCR mixture (50 μl total volume) contained: 1× AccuPrime PCR Buffer II (containing MgCl_2_, nucleotides), 200 nM primer (each), 5% DMSO, 1 U AccuPrime Taq DNA polymerase (Invitrogen, Carlsbad, CA, United States), and 100 ng template DNA. The amplification was performed in Bio-Rad C-1000 cycler (Bio-Rad, Hercules, CA, United States) using a touch down protocol consisting of a hold at 94°C for 5 min followed by 10 cycles of denaturing at 94°C for 60 s, annealing at 66–56°C (each cycle the temperature decreased by 1°C) for 50 s, and extension at 72°C for 30 s, then 30 cycles of 94°C for 60 s, 56°C for 50 s, and 72°C for 30 s, and final extension at 72°C for 5 min.

The PCR products were purified by QIAquick^®^ PCR Purification Kit (Qiagen, Hilden, Germany), cloned with pGEM^®^-T Easy Vestor System (Promega, Madison, WI, United States) according to the manufacturer’s protocol, and transformed into *Escherichia coli* JM109. The transformant colonies were transferred to 20 μl of sterile water and lysed for 5 min at 95°C. A 5 μl aliquot was transferred to a PCR mixture containing in 25 μl total volume: 1× DreamTaq Buffer, 400 nM pUC-M13f and pUC-M13r primers (Promega), 200 μM dNTP, 1 U DreamTaq DNA Polymerase (Fermentas, Waltham, MA, United States). The cloned inserts were amplified using the PCR protocol: initiation at 94°C for 5 min, 35 cycles of denaturing at 94°C for 60 s, annealing at 54°C for 50 s, extension at 72°C for 30 s, and final extension at 72°C for 5 min. The PCR products were purified using QIAquick^®^ PCR Purification Kit (Qiagen) and sequenced by Macrogen Europe Laboratory (Amsterdam, Netherlands). The sequences were edited in Chromas Lite Software (Technelysium, Brisbane, QLD, Australia), converted to the amino acid sequences in BioEdit Sequence Alignment Editor v. 7.0.5.3^2^, aligned with Muscle v3.6 (Edgar, 2004) and analyzed in Mothur 1.39.5 (Schloss et al., 2009). Fifty sequences of each treatment were selected for the libraries, which were compared between the sites and treatments by Libshuff method (Singleton et al., 2001) implemented in Mothur.

### Quantitative PCR

Primers were eub338f (5′-ACTCCTACGGGAGGCAGCAG-3′) (Lane, 1991) and eub518r (5′-ATTACCGCGGCTGCTGG-3′) (Muyzer et al., 1993) amplifying a 197 bp fragment of the 16S rRNA gene from *Bacteria*, act235f (5′-CGCGGCCTATCAGCTTGTTG-3′) (Stach et al., 2003) and eub518r, yielding a 280 bp product, for *Actinobacteria*. The reactions were done on a StepOnePlus Real-Time PCR System (Applied Biosystems, Foster City, CA, United States) using 96-well plates with GoTaq qPCR Master Mix (Promega) containing SYBR Green as a double-stranded DNA binding dye. The reaction mixture contained in a total volume of 15 μl: 1× GoTaq qPCR Master Mix, 0.2 μM primers, and 1 ng diluted DNA sample. For both targets the PCR cycling protocol consisted of initial denaturation at 95°C for 10 min, followed by 45 cycles of 95°C for 15 s, 60°C for 30 s and 72°C for 30 s. Melting curves were recorded to ensure qPCR specificity. Baseline and threshold calculations were performed with the StepOne v. 2.2.2 software. The inhibition was tested by serial DNA dilutions from each site. All qPCR measurements were done in duplicates. The qPCR standard was prepared by cloning the fragments of the target gene from *Streptomyces europaeiscabiei* DMS 41802 as described previously (Sagova-Mareckova et al., 2015).

### Illumina Amplicon Sequencing

Two randomly selected replicates of each treatment were chosen for sequencing (the number of sequenced replicates was limited to two for economic reasons after observation of low variability of replicates in qPCR). The environmental DNA samples were diluted to approximately 30 ng μl^-1^ in a volume of 50 μl and transferred to transport tubes GenTegra DNA (GenTegra, Pleasanton, CA, United States). The samples were thoroughly mixed with the protective medium, and dried in a vacuum evaporator at 37°C for 240 min. Amplification of bacterial 16S rRNA gene fragment including the variable region V4 using universal primers with overhang adapters CS1-515F (5′-ACACTGACGACATGGTTCTACAGTGCCAGCMGCCGCGGTAA-3′) and CS2-806R 5′-TACGGTAGCAGAGACTTGGTCTGGACTACHVGGGTWTCTAAT-3′) (Caporaso et al., 2011), construction of libraries, and sequencing by Illumina MiSeq (Illumina, San Diego, CA, United States) were done at the DNA Services Facility, Research Resources Centre University of Illinois (Chicago, IL, United States). The resulting paired sequence reads were merged, filtered, aligned using reference alignment from the Silva database (Quast et al., 2013), and chimera checked using integrated Vsearch tool (Rognes et al., 2016) according to the MiSeq standard operation procedure (Miseq SOP, February 2018; Kozich et al., 2013) in Mothur v1.39.5 (Schloss et al., 2009). A taxonomic assignment of sequence libraries was performed in Mothur using the Silva Small Subunit rRNA Database, release 132 (Yilmaz et al., 2014) adapted for use in Mothur^3^ as the reference database. Sequences of plastids, mitochondria, and those not classified in the domain *Bacteria* were discarded. The sequence library was clustered into OTUs using the Uparse pipeline in Usearch v10.0.240 software (Edgar, 2013), and the OTU table was further processed using tools implemented in the Mothur. Distance matrices describing the differences in community composition between individual samples were calculated using the Yue-Clayton theta calculator (Yue and Clayton, 2005). Analysis of molecular variance (AMOVA; Martin, 2002) was based on a matrix of Yue-Clayton theta distances. Metastats (White et al., 2009) and Lefse (Segata et al., 2011) analyses were used to detect differentially represented OTUs. A Maximum-likelihood phylogram of the OTU representative sequences was constructed using FastTree 2 (Price et al., 2010). Rarefaction curves were used as a measure of diversity because Illumina sequencing shows only relative rather than absolute quantity of individual taxa measured and the total number of OTUs in the community cannot be determined (Edgar, 2013). Figures were created using the Interactive Tree of Life online tool^4^ (Letunic and Bork, 2016), and Inkscape (v0.92^5^).

### Statistical Analyses

Analysis of variance (ANOVA) tests were performed for respiration, copy numbers of bacterial and *Actinobacteria* 16S rRNA genes to determine differences between the treatments. *P*-values for the pairwise comparison were adjusted for multiple comparison problems with the help of the Max-abs-t-distribution method (Bretz et al., 2015). Yue-Clayton theta distances of bacterial communities were used in the testing. Differences within and between groups were determined by Adonis test, which is a permutational multivariate analysis of variance using distance matrices (McArdle and Anderson, 2001). Dispersion within groups was determined by test of multivariate dispersions using dissimilarity measures (Gijbels and Omelka, 2013). All statistical calculations were done in the R computing environment (R Core Team, 2017).

### Accession Numbers

The partial cellobiohydrolase gene sequences reported in this paper have been deposited in GenBank under accession no. MF621345 – MF621548. The Illumina MiSeq 16S rRNA gene amplicon sequences have been deposited in the NCBI Sequence Read Archive^6^ as BioProject PRJNA397131.

## Results

### Original Soils

**FIGURE 1:**
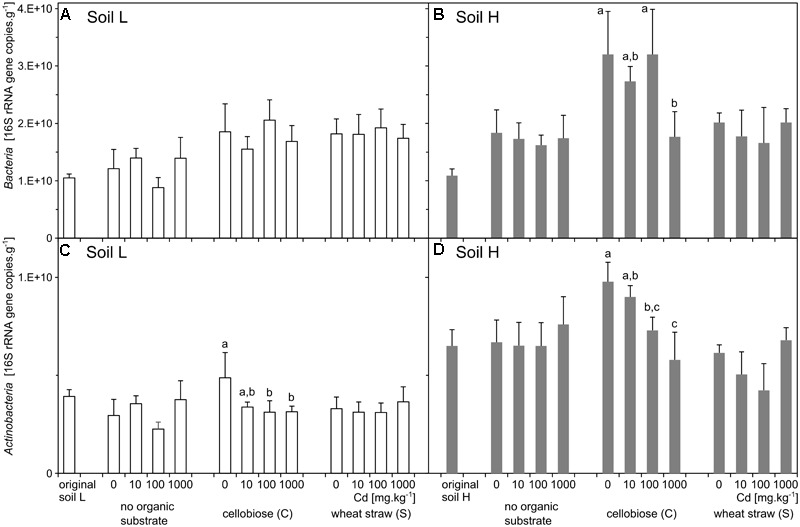
Abundances of total bacteria **(A,B)** and actinobacteria **(C,D)**. 16S rRNA gene copy numbers according to quantitative real-time PCR in original soils and treatments (*n* = 5) of the less contaminated soil L (white), and the more contaminated soil H (gray). Different letters indicate significant differences between cadmium treatments tested within the subset of samples supplemented with the same organic substrate.

**FIGURE 2:**
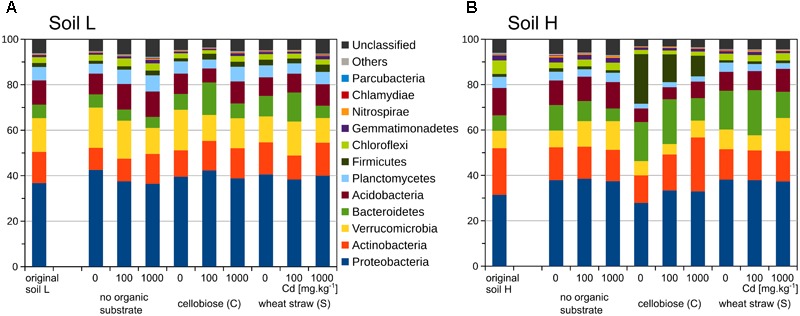
Proportions of bacterial phyla in the bacterial 16S rRNA gene amplicon libraries (*n* = 2) from original soils and treatments of the less contaminated soil L **(A)**, and the more contaminated soil H **(B)**.

The two original soils differed significantly by the content of toxic elements after the long term exposure to contamination. The soil with low contamination (L) had approximately half of the contaminating Pb, Zn, As, and Cd than the soil with high contamination (H) (Table 2). The quantity of total bacteria did not differ between the two original soils (Figures 1A,B), but quantity of actinobacteria was about 40% higher in H soil than L soil (*p* < 0.001, Figures 1C,D). The proportion of phyla was relatively similar in the two soils, only *Actinobacteria* and *Chloroflexi* were slightly higher in H soil (Figure 2).

### Respiration

**FIGURE 3:**
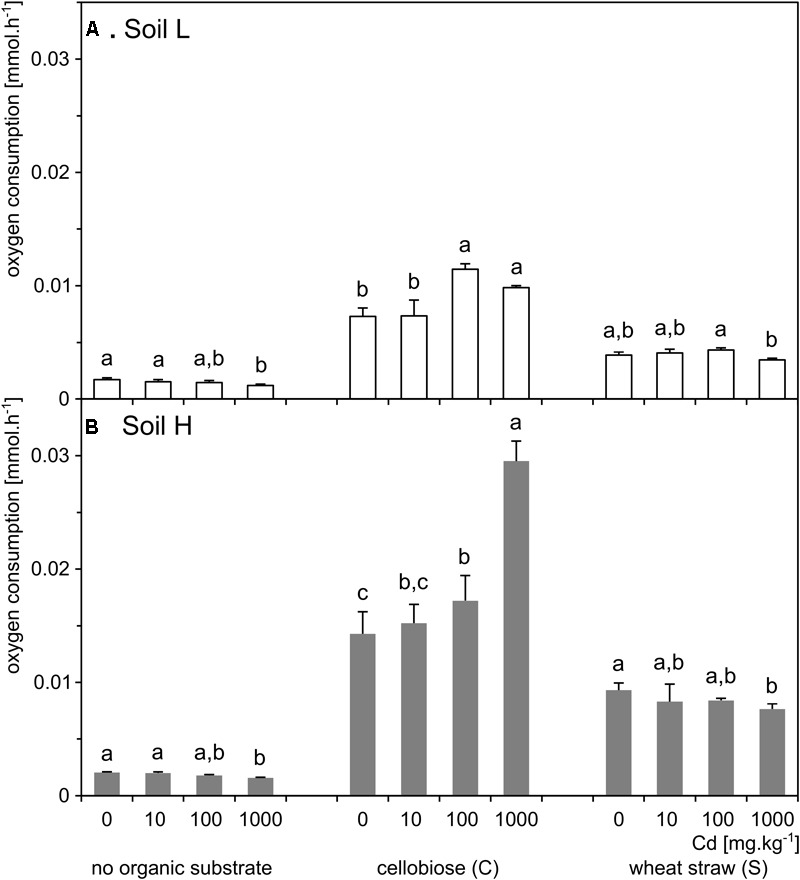
Respiration rates of soil microbial communities in the samples of the less contaminated soil L (**A**; white) and the more contaminated soil H (**B**; gray) supplemented with organic substrates and cadmium (*n* = 5). Letters indicate significant differences between cadmium treatments tested within the subset of samples supplemented with the same organic substrate.

Respiration of the soil H was significantly higher than of the soil L (*p* < 0.001; Figure 3). The respiration increased 5–10 times in treatments supplemented with organic carbon substrates in both soils, but it increased several times more in the H soil. In both soils, respiration was significantly affected by interaction between effects of cadmium and substrate (*p* < 0.001). Differences between cadmium doses within organic substrate treatments were similar in the two soils. However, in L soil, both cadmium and substrate additions had a total significant effect on respiration (*p* < 0.001), while in H soil, only the effect of added organic carbon substrate (*p* < 0.001) was significant (Figure 3 and Supplementary Table S1).

### Quantity of Bacteria and Actinobacteria

The effect of treatments on bacteria and actinobacteria quantities differed in the two soils. In the treatments of H soil, the numbers of bacteria were influenced by interaction between effects of cadmium and substrate (*p* = 0.022) and the interaction was due to the treatment by cadmium 1,000 mg.kg^-1^, which significantly decreased bacteria number in cellobiose treatment. The substrate alone also increased numbers of bacteria (*p* < 0.001), particularly after addition of cellobiose (Figures 1A,B and Supplementary Table S1). The numbers of actinobacteria were also influenced by interaction between effect of cadmium and substrate (*p* < 0.001). That significant effect was mostly due to treatment with cadmium 1,000 mg.kg^-1^, which increased numbers of bacteria in the straw treatment. However, both cadmium and substrate significantly influenced numbers of actinobacteria also separately (*p* = 0.004; *p* < 0.001, respectively). In the treatments of L soil, the numbers of bacteria were not influenced by interaction between the effects of cadmium and substrate and only the effect of substrate was significant (*p* < 0.001). The numbers of actinobacteria were only marginally influenced by the interaction between the effects of cadmium and substrate (*p* = 0.021) and only the individual effect of cadmium was significant (*p* = 0.027) (Figures 1C,D and Supplementary Table S1).

### Cellobiohydrolase

Sequences of the gene coding for cellobiohydrolase differed significantly (Libshuff, *p* < 0.05) between the soil samples H0 and L0, which did not obtain any cadmium or organic substrate supplement and also between the samples with supplements of straw and cadmium in concentration 100 mg.kg^-1^ (Table 3).

**Table 3:** Comparison of cellobiohydrolase sequences between samples by Libshuff test.

	dCXYScore	Significance		dCXYScore	Significance
H0-L0	0.01233187	0.0002	HS100-LS100	0.00171228	0.2729
L0-H0	0.02299702	<0.0001	LS100-HS100	0.01491378	0.0002
L0-LS100	0.00139254	0.3119	H0-HS100	0.00121525	0.3241
LS100-L0	0.00141729	0.3753	HS100-H0	0.00248535	0.0885

*Site H: H0 – (no Cd, no substrate), HS100 – 100 mg/kg Cd, straw; site L: L0 – (no Cd, no substrate) LS100 – 100 mg/kg Cd, straw.*

### Bacterial Community

**FIGURE 4:**
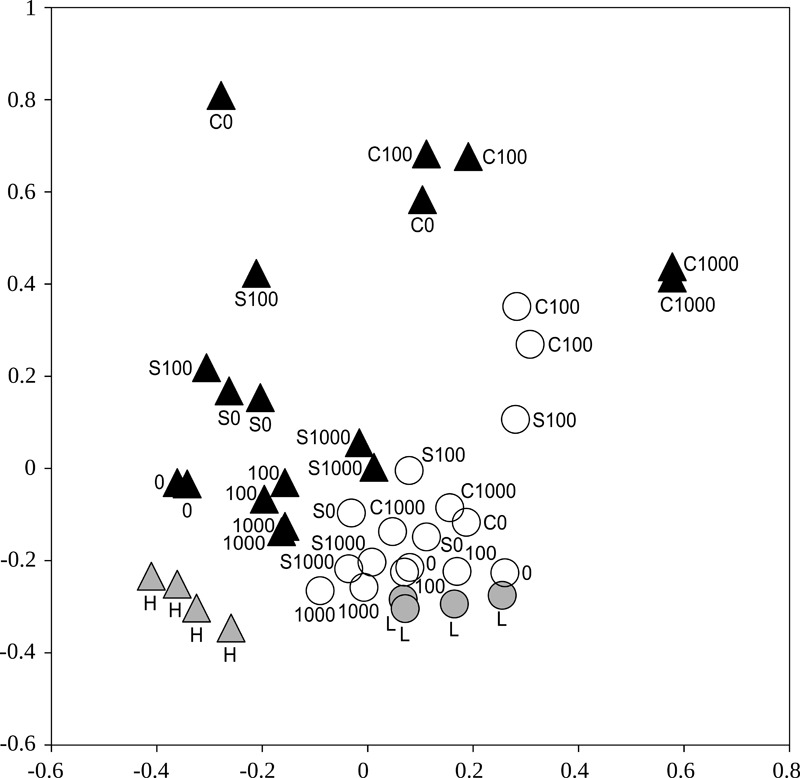
An ordination showing distances between the bacterial communities represented by 16S rRNA gene amplicon sequence libraries from samples of the less contaminated soil L (circles) and the more contaminated soil H (triangles) after treatments with cellobiose (C), wheat straw (S), and cadmium 0–1,000 mg kg^-1^. White and black symbols show soils after incubation in the respiration experiment (*n* = 2), gray symbols show the original soils (*n* = 4). Non-metric multidimensional scaling based on a matrix of Yue-Clayton theta distances.

Bacterial communities differed significantly between the two soils, when all treatments were included (Adonis, *p* < 0.001). Also, bacterial communities from the less contaminated soil L were more homogeneous compared to the more contaminated soil H (*p* disp < 0.001, Figure 4).

**FIGURE 5:**
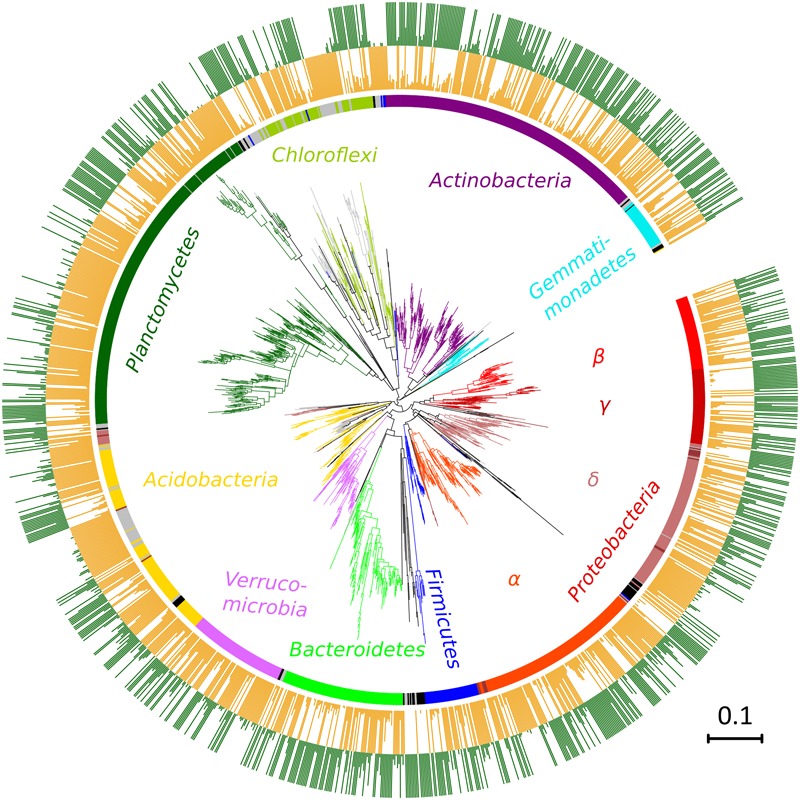
Phylogenetic classification of OTUs differing between the two original soils, the less contaminated soil L (orange) and the more contaminated soil H (green). Colors of the branches and inner circle indicate assignment to phyla, gray depicts unclassified OTUs, and black OTUs belonging to other less frequent phyla. Lengths of bars in the outer two rings show proportions of each OTU relative to its higher value in one of the two compared soils. A maximum-likelihood method from representative sequences of a subset of 1,594 OTUs.

**FIGURE 6:**
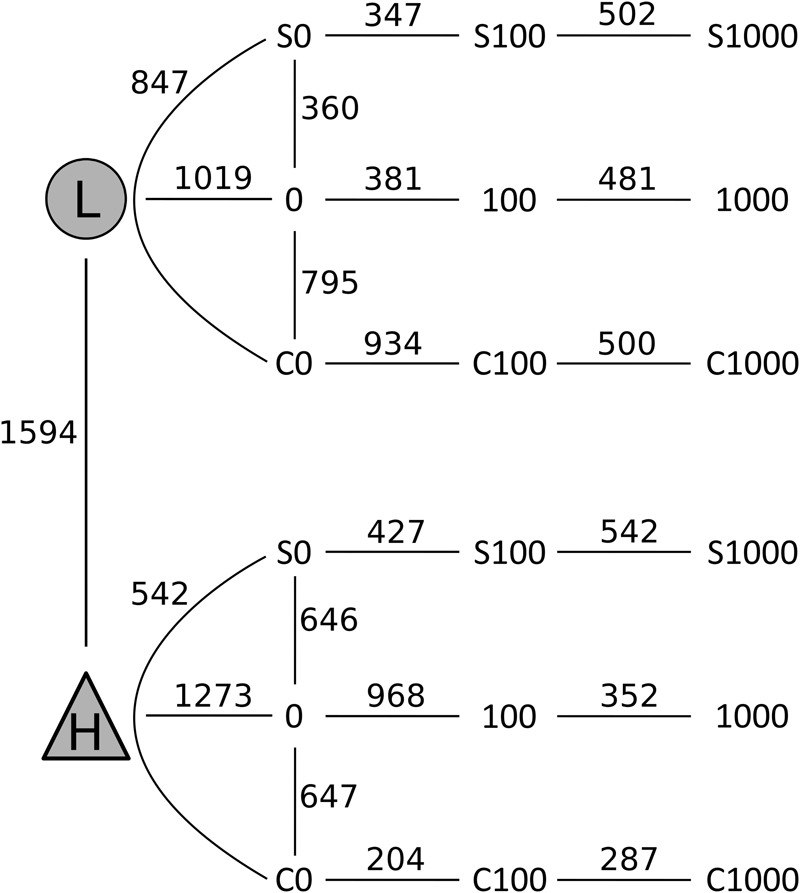
Changes in composition of bacterial communities from samples of the less contaminated soil L (circles) and the more contaminated soil H (triangles) after treatments with cellobiose (C), wheat straw (S), and cadmium 0–1,000 mg kg^-1^. Numbers on the lines connecting two treatments indicate the number of OTUs that significantly differed (*p* < 0.05) in pairwise comparisons using Metastats analysis.

After quality filtering, a total of 3,229,710 sequences were mapped to 16,311 OTUs defined at a 97% similarity level. The long-term effect of toxic elements contamination separated the two original soils by proportions of 1,594 OTUs (Metastats, *p* < 0.05) (Figure 5). After incubation in the short-term experiment, samples without cadmium or organic substrate supplement (L0, H0) differed significantly from the respective original soil in proportions of 1,019 OTUs in soil L, and in proportions of 1,273 OTUs in soil H. Those samples differed between the two soils by 815 OTUs (Figure 6). The numbers of OTUs reacting significantly to carbon substrates were 1114 (41 reacting to both of them) in L soil, and 1043 (250 reacting to both) in soil H (Metastats, *p* < 0.05; Figure 6). More specifically in treatments with cellobiose, relative proportion of OTUs did not change between treatments without cadmium and with cadmium 100 mg.kg^-1^ and 1,000 mg.kg^-1^ in L soil, while in H soil the relative proportions of respective OTUs decreased (Supplementary Figure S1).

The rarefaction curves showed that bacterial diversity was similar in H and L soils for both the original soils exposed to long-term contamination and samples from the experiment without cadmium or organic substrate. Generally in L soil, the diversity did not reflect the treatments, while in H soil the cellobiose treated samples had the lowest diversity. Yet, diversity was mostly higher in L soil than in H soil after all treatments (Supplementary Figure S2). The effect of cadmium on diversity was not significant but both carbon substrates affected diversity in both soils (AMOVA, L: *p* = 0.009, H: *p* < 0.001). However, in H soil the pairwise difference occurred between all treatments, while in L soil it was significant only between the control and addition of cellobiose.

The changes in bacterial communities after treatments occurred even on the high phylogenetic level of phyla (Figure 2). In particular, the relative proportion of *Firmicutes* increased in H soil after addition of cellobiose but not in L soil. On the level of *Firmicutes* families, in H soil *Clostridiaceae* dominated after addition of cellobiose in treatments without cadmium and with 100 mg.kg^-1^ of cadmium but *Planococcaceae* dominated in treatments with 1,000 mg.kg^-1^ of cadmium. in *Actinobacteria*, relative proportion of *Pseudonocardiaceae* is higher in the original soil H, while proportion of Trebon clade members (defined in Kopecky et al., 2011) is higher in the original soil L. Family of *Micrococcaceae* increased after addition of carbon substrate in both soils but in L soil decreased with additions of cadmium, while in H soil further increased even in highest cadmium dosage. In *Proteobacteria*, a similar situation occurred with *Enterobacteriaceae* and *Aeromonadaceae*, which increased by addition of cellobiose in H soil and *Pseudomonadaceae*, which also increased after addition of both carbon substrates but mostly in H soil. In H soil changes were observed also in decrease of relative abundance of *Verrucomicrobia* and increase of relative abundance of *Bacteroidetes*, of which particularly *Flavobacteria* increased in H soil after cellobiose additions (Supplementary Figures S3–S8).

## Discussion

The differences in bacterial diversity between the two soils occurred after addition of organic substrate and cadmium because the number of OTUs decreased in H soil and increased in L soil after treatments. In a similar experiment, microbial communities of soils differing in contamination remained similar after exposure to Cu and manure amendments (Brandt et al., 2010), while in another experiment changes in microbial communities after additions of different pollutants corresponded to the contamination history of the studied soils (Azarbad et al., 2015, 2016). Increased diversity in contaminated soils had been explained either by effects of toxic elements on diversification of the microbial populations due to resistance spreading or development of new chemical gradients, to which the microorganisms adapt (Ciarkowska et al., 2014; Yin et al., 2015). However, no clear explanation on why particular taxa respond to disturbance differently can be made as it is still only poorly understood how soil structure creates habitats and niches and how it regulates interactions (Schimel and Schaeffer, 2012).

More specifically in our study, significant changes in bacterial community structure occurred in cellobiose treatment. There, the phylum *Firmicutes* increased its proportion and became visibly sensitive to cadmium by changing the representation of *Clostridiaceae* family and a group of unclassified *Bacillales*. This may be related to both high growth rate with this carbon source and metal resistance specific to only some families within the phylum. Similar results showing higher resistance to toxic elements in *Firmicutes* were found in sites contaminated by Pb, Cu, and Zn (Ellis et al., 2003). *Actinobacteria* seemed relatively sensitive to cadmium in treatments with cellobiose because their numbers gradually decreased. High sensitivity of actinobacteria to toxic elements namely to Pb, Zn, Cr or Mn was observed previously (Yin et al., 2015) but opposite results were obtained by Li et al. (2015). A more detailed analysis showed that *Thermoleophilia, Propionibacteriales*, and *Pseudonocardiales* were rather tolerant to Cd, Pb, and Zn, while *Acidimicrobiales, Solirubrobacterales*, and *Frankiales* were not (Epelde et al., 2015). In our study, there was a strong increase of *Micrococcaceae* after organic substrate treatments with moderate tolerance to cadmium in H soil but not in L soil. *Proteobacteria* families *Pseudomonadaceae, Enterobacteriaceae* and *Aeromonadaceae* increased with organic substrate and seemed resistant to medium cadmium dosage. Yet, *Proteobacteria* seemed relatively sensitive to toxic elements stress in other studies (Li et al., 2015; Yin et al., 2015). *Bacteroidetes* increased after additions of both organic substrates and namely *Flavobacteria* increased after addition of cellobiose, which corresponds to their known decomposing abilities (Grondin et al., 2017). Three phyla *Proteobacteria, Firmicutes*, and *Actinobacteria* have been showed to possess both antibiotic and metal resistances (Li et al., 2017) so, we concluded that some taxa within those phyla might have benefited from this trait also at our site. It was shown previously that differences in resistance to metal contamination between various taxa may be related to changes of community permissiveness to plasmids carrying respective resistance genes. That occurs because although the bacterial communities possess required plasmids, there are differences in taxa, which carry particular resistances and above that type of disturbance and dose of contaminant strongly affected plasmid movement (Klümper et al., 2017). Finally, the variability in sequences of genes coding for cellobiohydrolase was specific for each of the two original soils and in the experiment it remained unchanged. Consequently it seems that cellobiohydrolase gene sequences are locally selected and decomposition of cellulose is carried out by the same taxa under treatments as in the original soils.

We demonstrated that differences between the two soils were observed in relative proportions of phyla but also in various lower taxonomic groups after both the long-term soil contamination and the short term treatments. That is supported by findings of Stefanowicz et al. (2012), who showed that community structure is a more sensitive determinant of disturbance than measurements of common respiration, biomass or catabolic abilities. It also agrees with several studies, which suggested that microbial communities respond to soil carbon with respect to their life history patterns that are deeply rooted in microbial phylogeny, i.e., that the functional groups appear at the level of families or phyla rather than species or genera (Allison and Martiny, 2008; Philippot et al., 2010; Schimel and Schaeffer, 2012; Shade et al., 2012). Consequently, we suggest that bacterial community composition reflects changes in ecosystem processes and may be used for bioindication.

The two soils had more than two centuries to gradually adapt to toxic elements pollution, so it was expected that their reaction to the additions of cadmium, cellobiose, and straw may be similar because of functional redundancy occurring in bacterial communities. However, higher respiration of the more contaminated soil H than less contaminated soil L after all treatments was in agreement with our alternative hypothesis of a stronger response of less healthy soils to secondary disturbance. Yet, that result disagreed with observations by other authors, who found decrease of respiration with increased primary contamination after secondary additions of pollutants and/or organic substrate (Brandt et al., 2010; Singh et al., 2014; Azarbad et al., 2015). In those studies, decrease in respiration was usually explained by decrease in microbial biomass and/or metabolic activities (Azarbad et al., 2013). That partially complies with our results, in which increase of respiration was paralleled with increase of bacteria numbers but mostly only after the treatment with cellobiose. However, some studies similarly as ours showed increased respiration after disturbance and reasoned that microorganisms in less polluted soils used a higher percentage of consumed carbon for assimilation and thus a smaller percentage was released as CO_2_ in dissimilation processes (Zhang et al., 2016). We similarly as Niemeyer et al. (2012) suggest that the difference in reactions of respiration to disturbance may be explained by changes in microbial community structure in combination with growth rate and resistance mechanisms specific for particular taxa after substrate additions. In our study, this explanation can be applied particularly to the response of actinobacteria, whose numbers were higher in the more contaminated soil H and also increased after all treatments. The observed adaptation of actinobacteria to toxic elements can be attributed to a relationship between toxic elements and antibiotics resistance. Actinobacteria are prominent producers of antibiotics, so they carry a wide range of resistances and it was previously demonstrated that those resistances are coupled or transferred on the same mobile genetic elements with heavy metal resistances (Seiler and Berendonk, 2012; Di Cesare et al., 2016). Besides the higher overall respiration observed in H soil, numbers of total bacteria and actinobacteria increased there after additions of both cadmium and substrate, while in the L numbers of bacteria increased slightly but numbers of actinobacteria remained the same. That again points to actinobacteria as a group with specific relationship to contamination in our soils.

## Conclusion

We found that the more contaminated soil was more sensitive to additional disturbance than the less contaminated soil. This applied for both response amplitude of respiration and changes in bacterial community, which further indicated differences in resilience and resistance in the two soils. More specifically, respiration in low contaminated soil was more resistant to disturbance. This was attributed to growth rates and resistance to toxic elements of particular bacterial taxa. That means, respiration can be used in determining resistance or resilience in soil health assessment but only after secondary disturbance because its amplitude changed only after treatments with organic substrates. That conclusion agrees with the previous suggestions by other authors. In the contrary, bacterial community and specifically *Actinobacteria* responded to toxic elements contamination by both their quantity and community composition in both long term contamination and short term experiment. So, we propose that this phylum has a potential for bioindication of soil health. Finally, the observed changes in the contaminated soil after over century long exposure to toxic elements contamination showed that even after a very long time soil contamination significantly affects both composition of bacterial community and respiration. Therefore, some of the replaced bacterial groups were not functionally redundant in the soil because changes in bacterial community resulted in changes of functioning. That has further consequences not only on local decomposition of organic matter but more importantly on carbon cycling.

## Author Contributions

MS-M designed the DNA based experiments and suggesting the methodological approach. JK conducted the bioinformatic analyses. PB designed the respiration experiments. MV and YS did the toxic element analyses. TV did the field sampling. PM did the qPCR and prepared Illumina sequencing. DR did the DNA extraction. MG designed the primers for cellobiohydrolase. MO conducted statistical analyses.

## Conflict of Interest Statement

The authors declare that the research was conducted in the absence of any commercial or financial relationships that could be construed as a potential conflict of interest.

## Footnotes

1 http://www.cazy.org/

2 http://www.mbio.ncsu.edu/BioEdit/bioedit.html

3 https://www.mothur.org/w/images/3/32/Silva.nr_v132.tgz

4 http://itol.embl.de

5 http://www.inkscape.org

6 www.ncbi.nlm.nih.gov/sra

## References

[B1] Allison S. D. Martiny J. B. H. (2008). Colloquium paper: resistance, resilience, and redundancy in microbial communities. *Proc. Natl. Acad. Sci. U.S.A.* 105(Suppl.) 11512–11519. 10.1073/pnas.0801925105 18695234 PMC2556421

[B2] Ashelford K. E. Weightman A. J. Fry J. C. (2002). PRIMROSE: a computer program for generating and estimating the phylogenetic range of 16S rRNA oligonucleotide probes and primers in conjunction with the RDP-II database. *Nucleic Acids Res.* 30 3481–3489. 10.1093/nar/gkf450 12140334 PMC137075

[B3] Azarbad H. Nikliñska M. Nikiel K. van Straalen N. M. Röling W. F. M. (2015). Functional and compositional responses in soil microbial communities along two metal pollution gradients: does the level of historical pollution affect resistance against secondary stress? *Biol. Fertil. Soils* 51 879–890. 10.1007/s00374-015-1033-0

[B4] Azarbad H. Niklinska M. Van Gestel C. A. M. Van Straalen N. M. Roling W. F. M. Laskowski R. (2013). Microbial community structure and functioning along metal pollution gradients. *Environ. Toxicol. Chem.* 32 1992–2002. 10.1002/etc.2269 23637098

[B5] Azarbad H. van Straalen N. M. Laskowski R. Nikiel K. Röling W. F. M. Nikliñska M. (2016). Susceptibility to additional stressors in metal-tolerant soil microbial communities from two pollution gradients. *Appl. Soil Ecol.* 98 233–242. 10.1016/j.apsoil.2015.10.020

[B6] Brandt K. K. Frandsen R. J. N. Holm P. E. Nybroe O. (2010). Development of pollution-induced community tolerance is linked to structural and functional resilience of a soil bacterial community following a five-year field exposure to copper. *Soil Biol. Biochem.* 42 748–757. 10.1016/j.soilbio.2010.01.008

[B7] Bretz F. Hothorn T. Westfall P. (2015). *Multiple Comparisons Using R.* (New York, NY: Chapman and Hall/CRC) 205.

[B8] Caporaso J. G. Lauber C. L. Walters W. A. Berg-Lyons D. Lozupone C. A. Turnbaugh P. J. (2011). Global patterns of 16S rRNA diversity at a depth of millions of sequences per sample. *Proc. Natl. Acad. Sci.* 108 4516–4522. 10.1073/pnas.1000080107 20534432 PMC3063599

[B9] Ciarkowska K. Sołek-Podwika K. Wieczorek J. (2014). Enzyme activity as an indicator of soil-rehabilitation processes at a zinc and lead ore mining and processing area. *J. Environ. Manage.* 132 250–256. 10.1016/j.jenvman.2013.10.022 24321285

[B10] Di Cesare A. Eckert E. M. D’Urso S. Bertoni R. Gillan D. C. Wattiez R. (2016). Co-occurrence of integrase 1 antibiotic and heavy metal resistance genes in municipal wastewater treatment plants. *Water Res.* 94 208–214. 10.1016/j.watres.2016.02.049 26945964

[B11] Edgar R. C. (2004). MUSCLE: multiple sequence alignment with high accuracy and high throughput. *Nucleic Acids Res.* 32 1792–1797. 10.1093/nar/gkh340 15034147 PMC390337

[B12] Edgar R. C. (2013). UPARSE: highly accurate OTU sequences from microbial amplicon reads. *Nat. Methods* 10 996–998. 10.1038/nmeth.2604 23955772

[B13] Ellis R. J. Morgan P. Weightman A. J. Fry J. C. (2003). Cultivation-dependent and-independent approaches for determining bacterial diversity in heavy-metal-contaminated soil. *Appl. Environ. Microbiol.* 69 3223–3230. 10.1128/AEM.69.6.3223 12788719 PMC161537

[B14] Epelde L. Lanzén A. Blanco F. Urich T. Garbisu C. (2015). Adaptation of soil microbial community structure and function to chronic metal contamination at an abandoned Pb-Zn mine. *FEMS Microbiol. Ecol.* 91 1–11. 10.1093/femsec/fiu007 25764532

[B15] Gijbels I. Omelka M. (2013). Testing for homogeneity of multivariate dispersions using dissimilarity measures. *Biometrics* 69 137–145. 10.1111/j.1541-0420.2012.01797.x 23002793

[B16] Griffiths B. S. Philippot L. (2013). Insights into the resistance and resilience of the soil microbial community. *FEMS Microbiol. Rev.* 37 112–129. 10.1111/j.1574-6976.2012.00343.x 22568555

[B17] Grondin J. M. Tamura K. Déjean G. Abbott D. W. Brumer H. (2017). Polysaccharide utilization loci: fueling microbial communities. *J. Bacteriol.* 199 e860–16. 10.1128/JB.00860-16 28138099 PMC5512228

[B18] Hartmann M. Niklaus P. A. Zimmermann S. Schmutz S. Kremer J. Abarenkov K. (2014). Resistance and resilience of the forest soil microbiome to logging-associated compaction. *ISME J.* 8 226–244. 10.1038/ismej.2013.141 24030594 PMC3869018

[B19] Jin Z. Li Z. Li Q. Hu Q. Yang R. Tang H. (2014). Canonical correspondence analysis of soil heavy metal pollution, microflora and enzyme activities in the Pb–Zn mine tailing dam collapse area of sidi village, SW China. *Environ. Earth Sci.* 73 267–274. 10.1007/s12665-014-3421-4

[B20] Klümper U. Dechesne A. Riber L. Brandt K. K. Gülay A. Sørensen S. J. (2017). Metal stressors consistently modulate bacterial conjugal plasmid uptake potential in a phylogenetically conserved manner. *ISME J.* 11 152–165. 10.1038/ismej.2016.98 27482924 PMC5097465

[B21] Kopecky J. Kyselkova M. Omelka M. Cermak L. Novotna J. Grundmann G. L. (2011). Actinobacterial community dominated by a distinct clade in acidic soil of a waterlogged deciduous forest. *FEMS Microbiol. Ecol.* 78 386–394. 10.1111/j.1574-6941.2011.01173.x 22092176

[B22] Kozich J. J. Westcott S. L. Baxter N. T. Highlander S. K. Schloss P. D. (2013). Development of a dual-index sequencing strategy and curation pipeline for analyzing amplicon sequence data on the miseq illumina sequencing platform. *Appl. Environ. Microbiol.* 79 5112–5120. 10.1128/AEM.01043-13 23793624 PMC3753973

[B23] Lane D. J. (1991). “16S/23S rRNA Sequencing,” in *Nucleic Acid Techniques in Bacterial Systematics* eds Stackebrandt E. Goodfellow M. (Chichester: John Wiley and Sons) 115–175.

[B24] Letunic I. Bork P. (2016). Interactive tree of life (iTOL) v3: an online tool for the display and annotation of phylogenetic and other trees. *Nucleic Acids Res.* 44 W242–W245. 10.1093/nar/gkw290 27095192 PMC4987883

[B25] Li J. Hu H. W. Ma Y. B. Wang J. T. Liu Y. R. He J. Z. (2015). Long-term nickel exposure altered the bacterial community composition but not diversity in two contrasting agricultural soils. *Environ. Sci. Pollut. Res.* 22 10496–10505. 10.1007/s11356-015-4232-1 25728202

[B26] Li J. Zheng Y. M. Liu Y. R. Ma Y. B. Hu H. W. He J. Z. (2014). Initial copper stress strengthens the resistance of soil microorganisms to a subsequent copper stress. *Microb. Ecol.* 67 931–941. 10.1007/s00248-014-0391-8 24682341

[B27] Li L. G. Xia Y. Zhang T. (2017). Co-occurrence of antibiotic and metal resistance genes revealed in complete genome collection. *ISME J.* 11 651–662. 10.1038/ismej.2016.155 27959344 PMC5322307

[B28] Lombard V. Golaconda Ramulu H. Drula E. Coutinho P. M. Henrissat B. (2014). The carbohydrate-active enzymes database (CAZy) in 2013. *Nucleic Acids Res.* 42 D490–D495. 10.1093/nar/gkt1178 24270786 PMC3965031

[B29] Martin A. P. (2002). Phylogenetic approaches for describing and comparing the diversity of microbial phylogenetic approaches for describing and comparing the diversity of microbial communities. *DNA Seq.* 68 3673–3682. 10.1128/AEM.68.8.3673 12147459 PMC124012

[B30] McArdle B. H. Anderson M. J. (2001). Fitting multivariate models to community data: a comment on distance-based redundancy analysis. *Ecology* 82 290–297. 10.1890/0012-96582001082

[B31] Muhlbachova G. Sagova-Mareckova M. Omelka M. Szakova J. Tlustos P. (2015). The influence of soil organic carbon on interactions between microbial parameters and metal concentrations at a long-term contaminated site. *Sci. Total Environ.* 502 218–223. 10.1016/j.scitotenv.2014.08.079 25260167

[B32] Muyzer G. De Waal E. C. Uitterlinden A. G. (1993). Profiling of complex microbial populations by denaturing gradient gel electrophoresis analysis of polymerase chain reaction-amplified genes coding for 16S rRNA. *Appl. Environ. Microbiol.* 59 695–700. 7683183 10.1128/aem.59.3.695-700.1993 PMC202176

[B33] Niemeyer J. C. Lolata G. B. Carvalho G. M. de Da Silva E. M. Sousa J. P. Nogueira M. A. (2012). Microbial indicators of soil health as tools for ecological risk assessment of a metal contaminated site in brazil. *Appl. Soil Ecol.* 59 96–105. 10.1016/j.apsoil.2012.03.019

[B34] Philippot L. Andersson S. G. E. Battin T. J. Prosser J. I. Schimel J. P. Whitman W. B. (2010). The ecological coherence of high bacterial taxonomic ranks. *Nat. Rev. Microbiol.* 8 523–529. 10.1038/nrmicro2367 20531276

[B35] Price M. N. Dehal P. S. Arkin A. P. (2010). FastTree 2 - approximately maximum-likelihood trees for large alignments. *PLoS One* 5:e9490. 10.1371/journal.pone.0009490 20224823 PMC2835736

[B36] Quast C. Pruesse E. Yilmaz P. Gerken J. Schweer T. Yarza P. (2013). The SILVA ribosomal RNA gene database project: improved data processing and web-based tools. *Nucleic Acids Res.* 41 D590–D596. 10.1093/nar/gks1219 23193283 PMC3531112

[B37] R Core Team. (2017). *R: A Language and Environment for Statistical Computing.* Vienna: R Found. Stat. Comput.

[B38] Rognes T. Flouri T. Nichols B. Quince C. Mahé F. (2016). VSEARCH: a versatile open source tool for metagenomics. *PeerJ* 4:e2584. 10.7717/peerj.2584 27781170 PMC5075697

[B39] Sagova-Mareckova M. Cermak L. Novotna J. Plhackova K. Forstova J. Kopecky J. (2008). Innovative methods for soil DNA purification tested in soils with widely differing characteristics. *Appl. Environ. Microbiol.* 74 2902–2907. 10.1128/AEM.02161-07 18344341 PMC2394906

[B40] Sagova-Mareckova M. Daniel O. Omelka M. Kristufek V. Divis J. Kopecky J. (2015). Determination of factors associated with natural soil suppressivity to potato common scab. *PLoS One* 10:e0116291. 10.1371/journal.pone.0116291 25612311 PMC4303419

[B41] Sagova-Mareckova M. Omelka M. Cermak L. Kamenik Z. Olsovska J. Hackl E. (2011). Microbial communities show parallels at sites with distinct litter and soil characteristics. *Appl. Environ. Microbiol.* 77 7560–7567. 10.1128/AEM.00527-11 21926225 PMC3209186

[B42] Schimel J. P. Schaeffer S. M. (2012). Microbial control over carbon cycling in soil. *Front. Microbiol.* 3:348 10.3389/fmicb.2012.00348 PMC3458434 23055998

[B43] Schloss P. D. Westcott S. L. Ryabin T. Hall J. R. Hartmann M. Hollister E. B. (2009). Introducing mothur: open-source, platform-independent, community-supported software for describing and comparing microbial communities. *Appl. Environ. Microbiol.* 75 7537–7541. 10.1128/AEM.01541-09 19801464 PMC2786419

[B44] Segata N. Izard J. Waldron L. Gevers D. Miropolsky L. Garrett W. S. (2011). Metagenomic biomarker discovery and explanation. *Genome Biol.* 12:R60. 10.1186/gb-2011-12-6-r60 21702898 PMC3218848

[B45] Seiler C. Berendonk T. U. (2012). Heavy metal driven co-selection of antibiotic resistance in soil and water bodies impacted by agriculture and aquaculture. *Front. Microbiol.* 3:399. 10.3389/fmicb.2012.00399 23248620 PMC3522115

[B46] Shade A. Peter H. Allison S. D. Baho D. L. Berga M. Bürgmann H. (2012). Fundamentals of microbial community resistance and resilience. *Front. Microbiol.* 3:417. 10.3389/fmicb.2012.00417 23267351 PMC3525951

[B47] Singh B. K. Quince C. Macdonald C. A. Khachane A. Thomas N. Al-Soud W. A. (2014). Loss of microbial diversity in soils is coincident with reductions in some specialized functions. *Environ. Microbiol.* 16 2408–2420. 10.1111/1462-2920.12353 24422656

[B48] Singleton D. R. Furlong M. Rathbun S. R. Whitman W. B. (2001). Environmental samples gene sequence libraries from quantitative comparisons of 16s rRNA. *Appl. Environ. Microbiol.* 67 4374–4376. 10.1128/AEM.67.9.4374-4376.2001 11526051 PMC93175

[B49] Stach J. E. M. Maldonado L. A. Ward A. C. Goodfellow M. Bull A. T. (2003). New primers for the class Actinobacteria: application to marine and terrestrial environments. *Environ. Microbiol.* 5 828–841. 10.1046/j.1462-2920.2003.00483.x 14510836

[B50] Stefanowicz A. M. Kapusta P. Szarek-Łukaszewska G. Grodzińska K. Niklińska M. Vogt R. D. (2012). Soil fertility and plant diversity enhance microbial performance in metal-polluted soils. *Sci. Total Environ.* 439 211–219. 10.1016/j.scitotenv.2012.09.030 23073370

[B51] Steinauer K. Chatzinotas A. Eisenhauer N. (2016). Root exudate cocktails: the link between plant diversity and soil microorganisms? *Ecol. Evol.* 6 7387–7396. 10.1002/ece3.2454 28725406 PMC5513276

[B52] Van Bruggen A. H. C. Semenov A. M. (2000). In search of biological indicators for soil health and disease suppression. *Appl. Soil Ecol.* 15 13–24. 10.1016/S0929-1393(00)00068-8

[B53] White J. R. Nagarajan N. Pop M. (2009). Statistical methods for detecting differentially abundant features in clinical metagenomic samples. *PLoS Comput. Biol.* 5:e1000352. 10.1371/journal.pcbi.1000352 19360128 PMC2661018

[B54] Yilmaz P. Parfrey L. W. Yarza P. Gerken J. Pruesse E. Quast C. (2014). The SILVA and “all-species living tree project (LTP)” taxonomic frameworks. *Nucleic Acids Res.* 42 D643–D648. 10.1093/nar/gkt1209 24293649 PMC3965112

[B55] Yin H. Niu J. Ren Y. Cong J. Zhang X. Fan F. (2015). An integrated insight into the response of sedimentary microbial communities to heavy metal contamination. *Sci. Rep.* 5 1–12. 10.1038/srep14266 26391875 PMC4585741

[B56] Yue J. C. Clayton M. K. (2005). A similarity measure based on species proportions. *Commun. Stat. Theory Methods* 34 2123–2131. 10.1080/STA-200066418 23236910

[B57] Zhang C. Nie S. Liang J. Zeng G. Wu H. Hua S. (2016). Effects of heavy metals and soil physicochemical properties on wetland soil microbial biomass and bacterial community structure. *Sci. Total Environ.* 55 785–790. 10.1016/j.scitotenv.2016.01.170 27046142

