# Supplementary material for: A Short-Term Response of Soil Microbial Communities to Cadmium and Organic Substrate Amendment in Long-Term Contaminated Soil by Toxic Elements
Source: Front Microbiol. 2018 Nov 20;9:2807. doi: 10.3389/fmicb.2018.02807 (PMC6256134; doi:10.3389/fmicb.2018.02807)
Supplement: Supplementary file 1 [file Data_Sheet_1.PDF]

Pavla Pruchova, Tomáš Větrovský, Marek Omelka, Michal Grunt, Yvona Smutna, Marek Vach, Petr Baldrian, Jan Kopecky, Marketa Sagova-Mareckova

The long and short term response of soil microbial communities to heavy metals in cultivated grassland

Supplementary material

Supplementary Figure S1. Phylogenetic classification of OTUs separating the control (green), and the treatments with cellobiose (orange), cellobiose + Cd, 100 mg kg-1 (light brown), and cellobiose + Cd, 1000 mg kg-1 (dark brown). Lenghts of the bars show proportion of each OTU relative to its maximum value among the compared treatments (full length). The phylogram was constructed by maximum likelihood method from representative sequences of a subset of 677 OTUs that significantly differed between the soils L and H or responded to the substrate or Cd treatments according to the Lefse analysis of the whole dataset.

A. Soil L

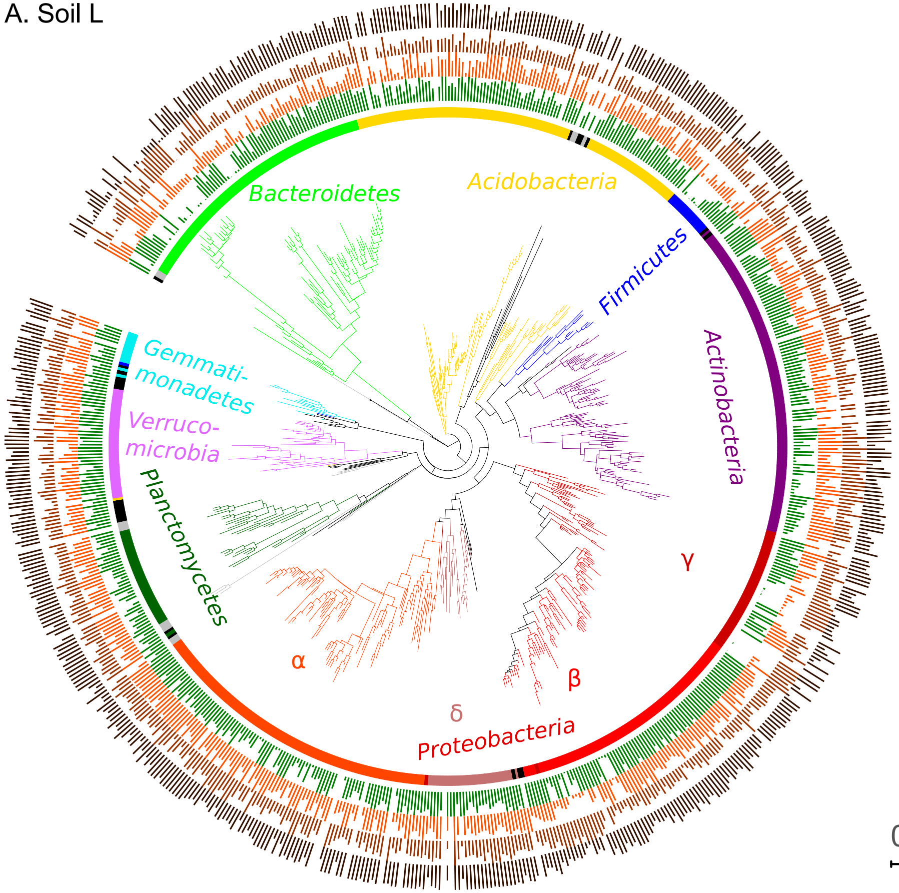

0.1

B. Soil H

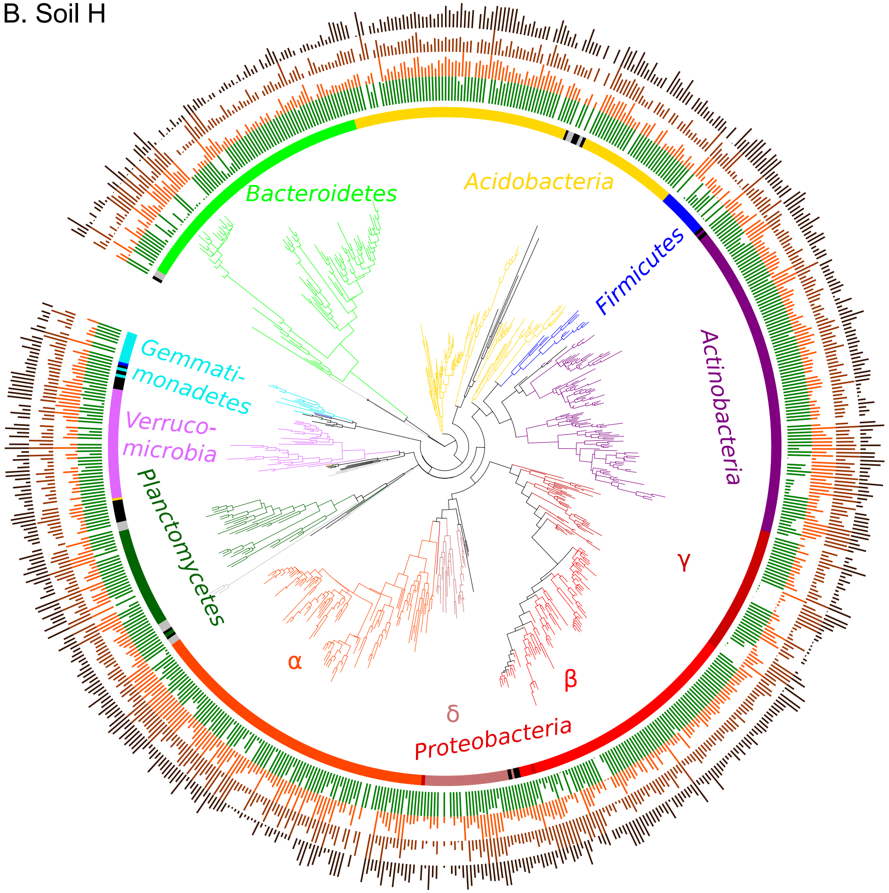

Supplementary Figure S2. Rarefaction analysis of sequence libraries from the treatments (n=2)

A. Soil L

B. Soil H

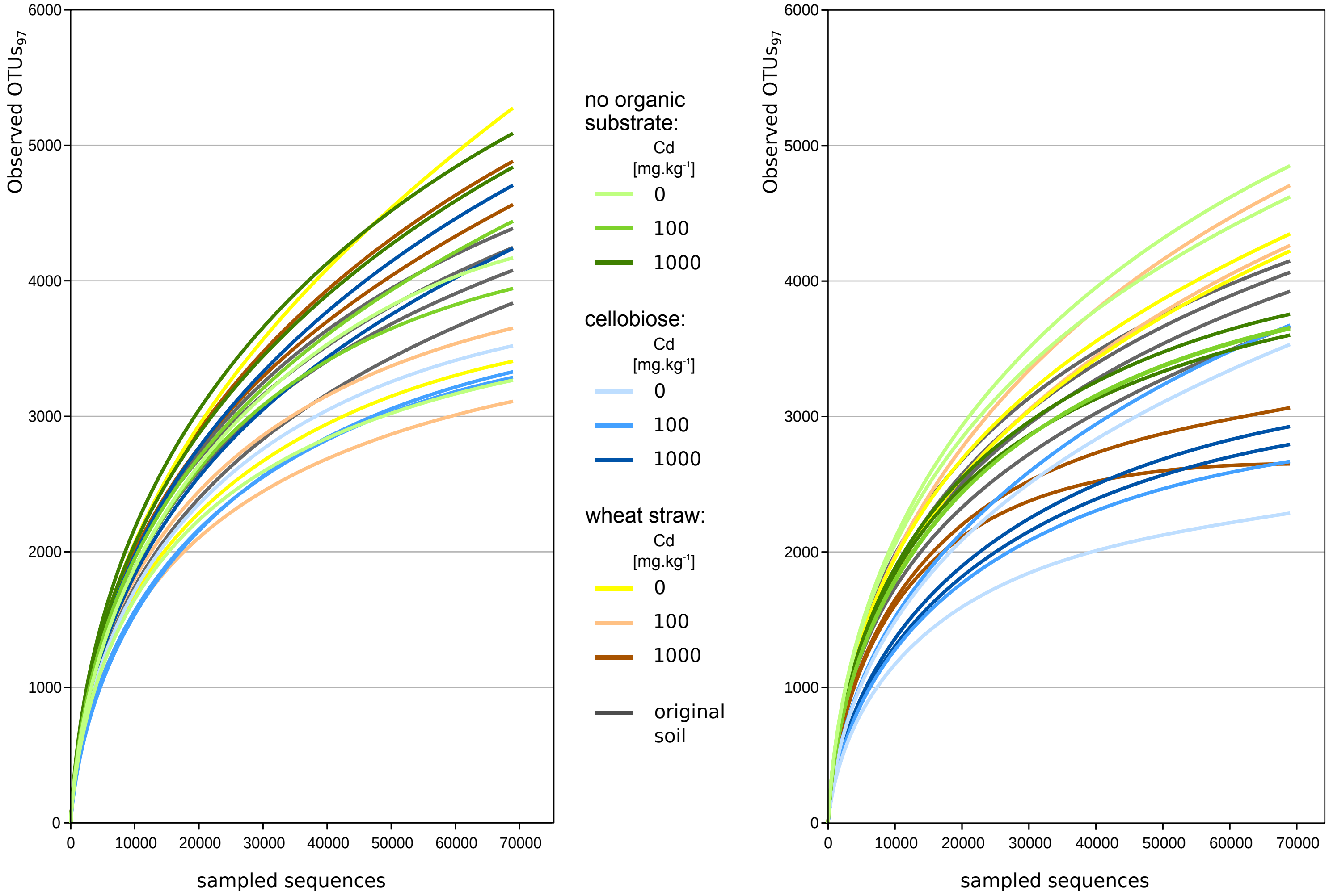

Supplementary Figure S3. Relative proportions of classes [%] within the phylum *Proteobacteria*

A.Soil L

B.Soil H

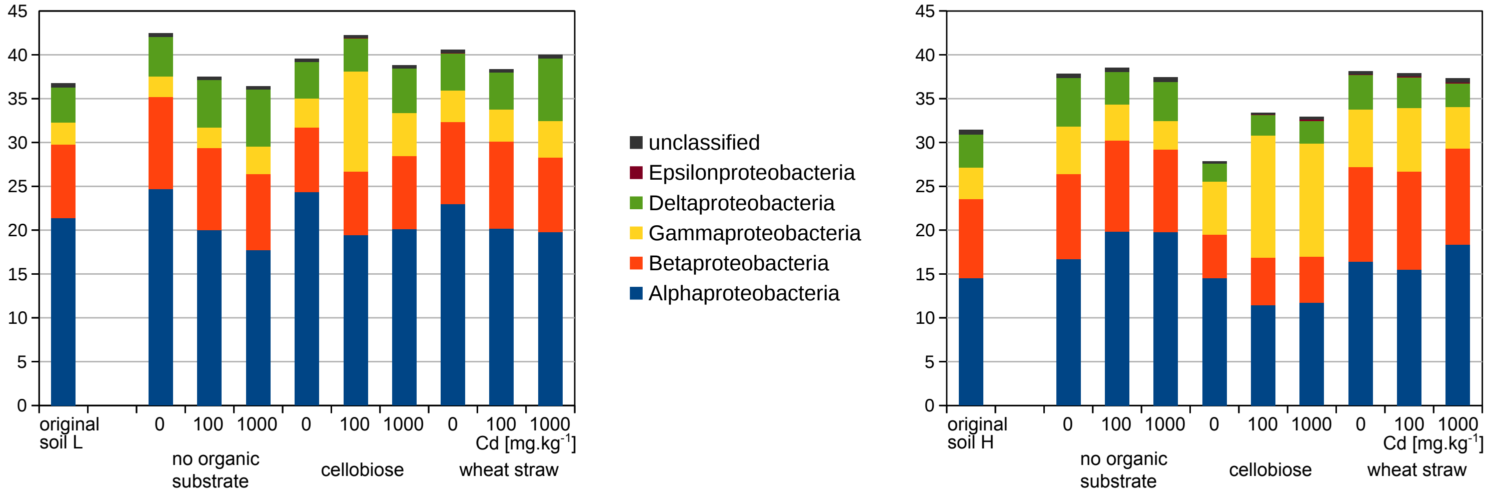

Supplementary Figure S4. Relative proportions of families [%] within the phylum *Proteobacteria*

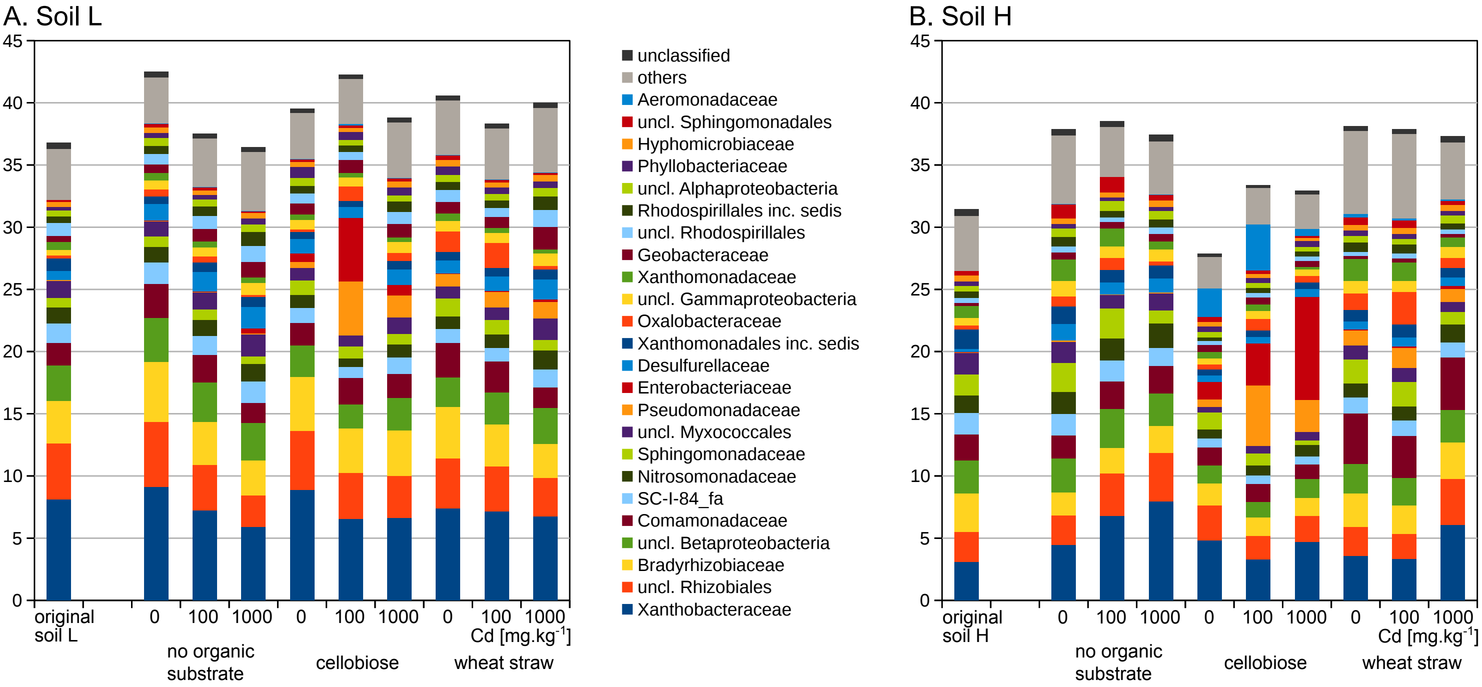

Supplementary Figure S5. Relative proportions of families [%] within the phylum *Actinobacteria*

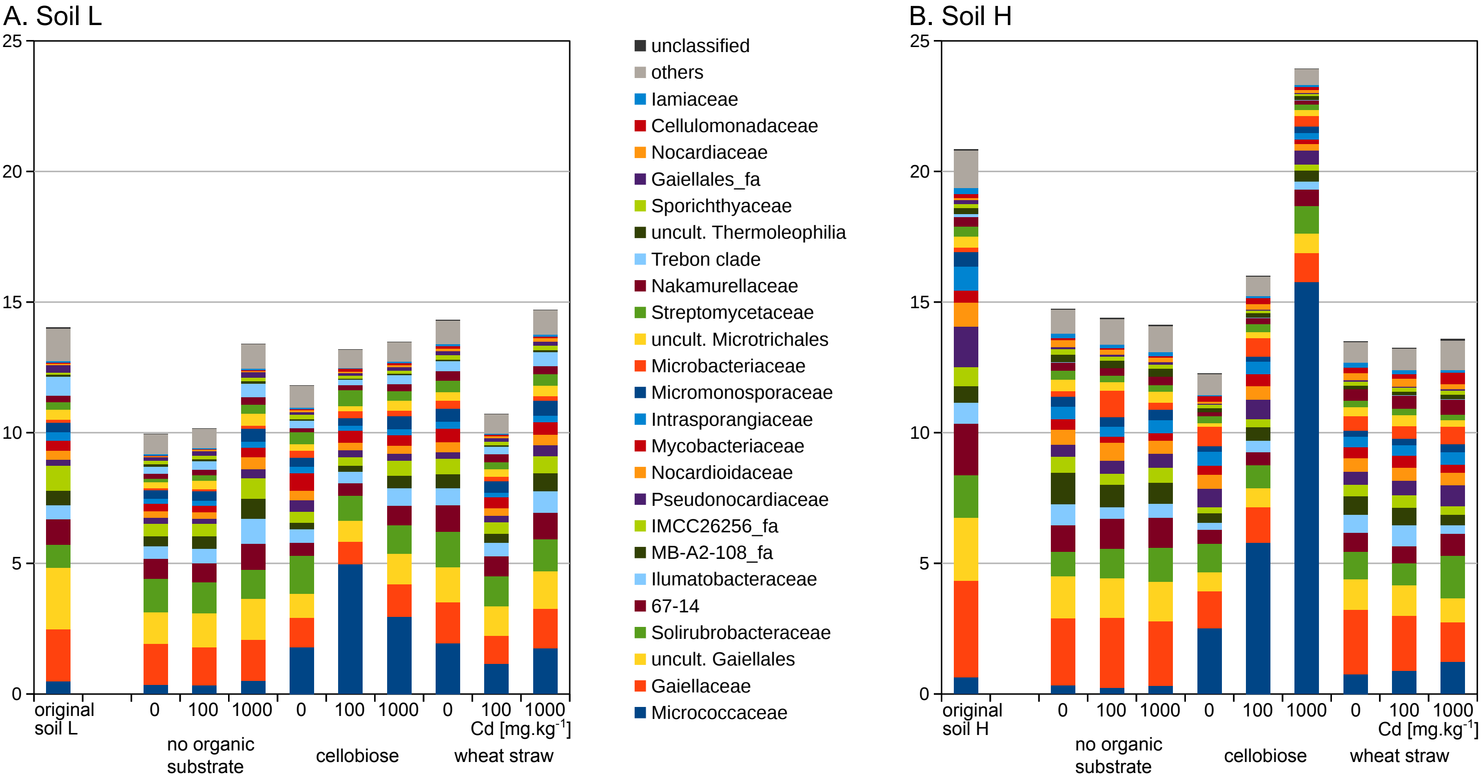

Supplementary Figure S6. Relative proportions of families [%] within the phylum *Firmicutes*

A. Soil L B. Soil H

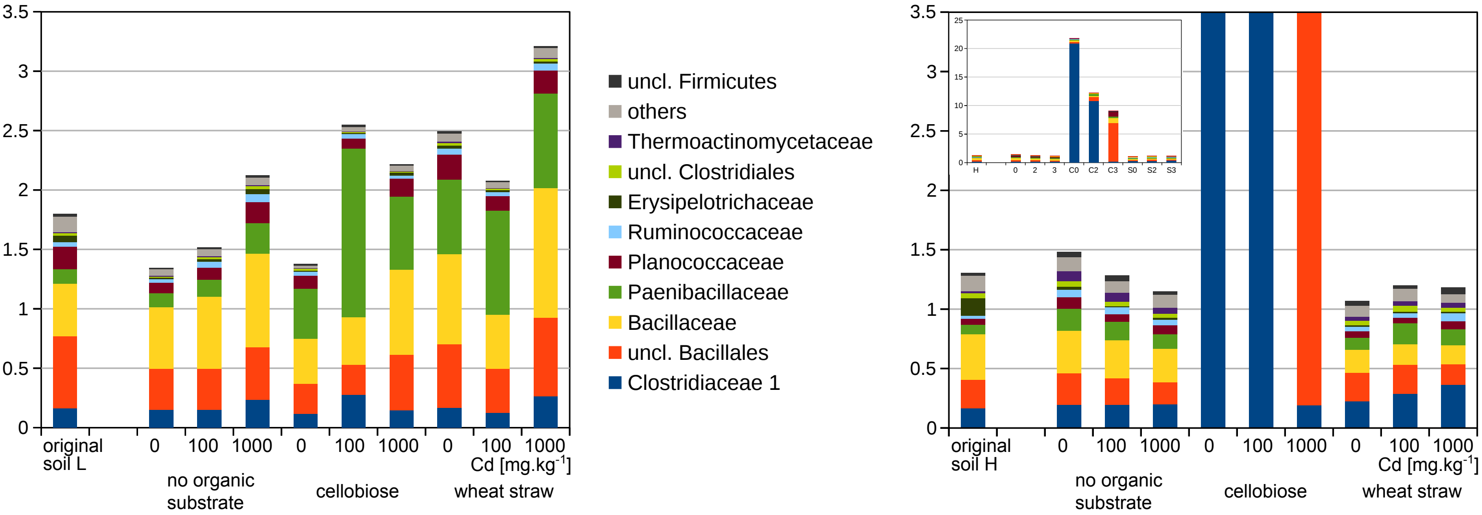

Supplementary Figure S7. Relative proportions of classes [%] within the phylum *Verrucomicrobia*

A. Soil L B. Soil H

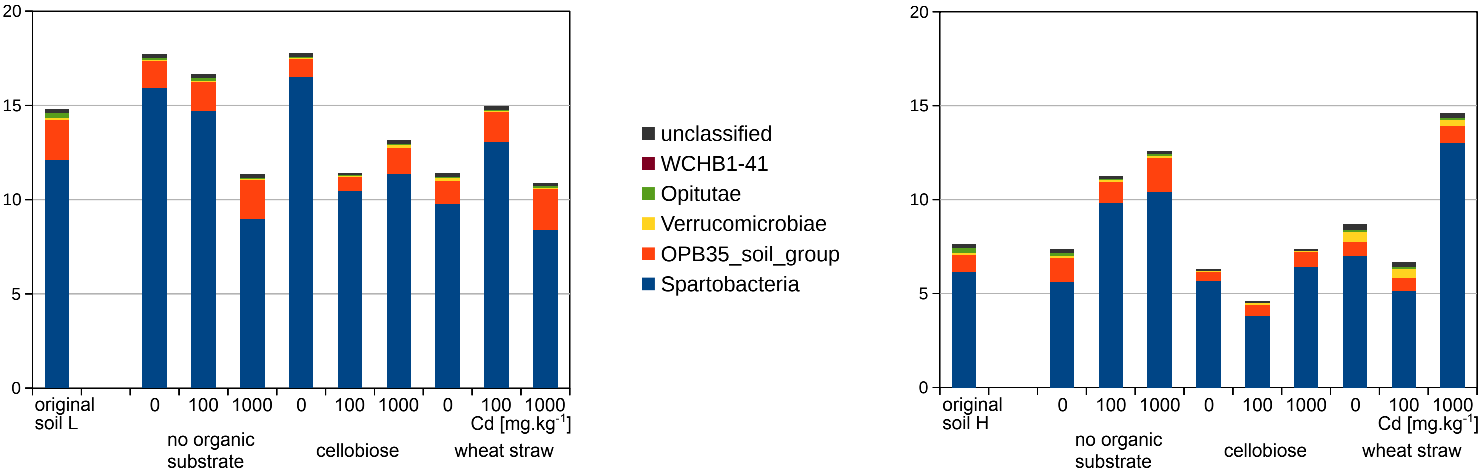

Supplementary Figure S8. Relative proportions of classes [%] within the phylum *Bacteroidetes*

A. Soil L B. Soil H

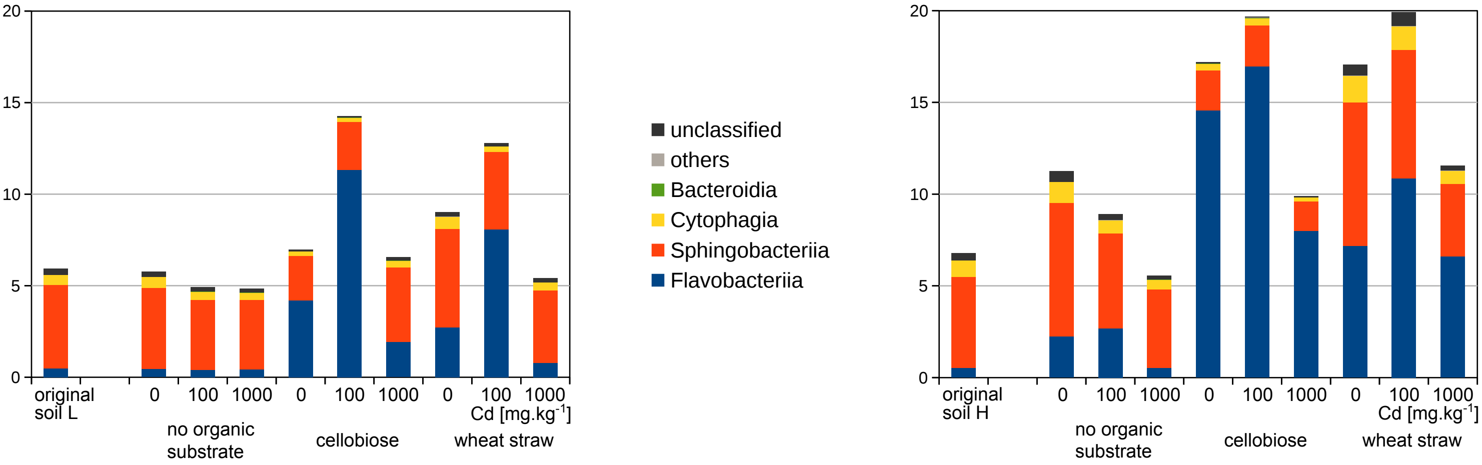

Supplementary Table 1. ANOVA results

A. Respiration

H soil

	Sum Sq	Df	F value	Pr(>F)
Substrate	47.094	2	2785.9	< 2.2e-16 ***
Cadmium	0.016	3	0.6307	0.599
Substrat:Cd	1.413	6	27.858	2.6e-13 ***
Residuals	0.363	43		

L soil

	Sum Sq	Df	F value	Pr(>F)
Substrate	28.388	2	1435.2	< 2.2e-16 ***
Cadmium	0.370	3	12.477	5.3e-06 ***
Substrat:Cd	0.799	6	13.472	1.6e-08 ***
Residuals	0.425	43		

B. Quantity of *Bacteria*

H soil

	Sum Sq	Df	F value	Pr(>F)
Substrate	984.34	2	22.137	6.1e-07 ***
Cadmium	117.09	3	1.7556	0.174
Substrate:Cd	381.72	6	2.8616	0.022 *
Residuals	778.13	35		

L soil

	Sum Sq	Df	F value	Pr(>F)
Substrate	366.24	2	17.074	6.7e-06 ***
Cadmium	1.27	3	0.0396	0.989
Substrate:Cd	131.76	6	2.0475	0.085
Residuals	375.39	35		

C. Quantity of *Actinobacteria*

H soil

	Sum Sq	Df	F value	Pr(>F)
Substrate	50.540	2	21.291	8.9e-07 ***
Cadmium	14.208	3	3.9902	0.015 *
Substrat:Cd	37.722	6	5.2970	<0.001 ***
Residuals	41.541	35		

L soil

	Sum Sq	Df	F value	Pr(>F)
Substrate	2.418	2	2.3418	0.111
Cadmium	5.361	3	3.4614	0.027 *
Substrate:Cd	9.025	6	2.914	0.021 *
Residuals	18.069	35		
